# Visible-light-driven radical Friedländer hetero-annulation of 2-aminoaryl ketone and α-methylene carbonyl compound via organic dye fluorescein through a single-electron transfer (SET) pathway

**DOI:** 10.1186/s13065-022-00910-1

**Published:** 2022-12-15

**Authors:** Farzaneh Mohamadpour

**Affiliations:** School of Engineering, Apadana Institute of Higher Education, Shiraz, Iran

**Keywords:** Photoexcited fluorescein, Visible light-mediated, Polysubstituted quinolines, Photochemical synthesis

## Abstract

The discoveries recommend that the photoinduced conditions of fluorescein-determined go about as impetus for photochemically combining polysubstituted quinolines in ethanol at room temperature under air environment by means of revolutionary Friedländer hetero-annulation of 2-aminoaryl ketone and α-methylene carbonyl compound. This study lays out an original capability for photochemically orchestrating fluorescein. This non-metallic organic dye is economically accessible and modest, producing great outcomes, accelerating the cycle, and achieving a high compound economy. The turnover number (TON) and turnover recurrence (TOF) of polysubstituted quinolines have been determined. This cycle will likewise run on a gram scale, demonstrating the chance of modern applications.

**Supplementary Information:**

The online version contains supplementary material available at 10.1186/s13065-022-00910-1.

## Introduction

Visible light, as a rich, effectively open, and sustainable clean energy source has ignited a ton of interest in supporting reactant natural blend reactions [1–9]. Noticeable light helped responses, in contrast with conventional manufacturing methods, meet the necessities of moderate reaction conditions, simplicity of activity, and ecological agreeableness. Most natural atoms, then again, can’t retain noticeable light, and the response must be supported by utilizing the right photocatalyst.

Organic dyes, which have shown identical photocatalytic interest in a couple of cycles, were utilized as an engaging choice to change metal buildings [10–13], attributable to their minimal expense and absence of poisonousness. Fluorescein has as of late been involved by Chu and colleagues for extremist buildup cyclization of benzimidazoles utilizing apparent light catalysis [14].

Quinolines have many pharmacological and natural impacts [15–43]. Various systems are accessible [44–57]. These treatments brought about a huge number of occurrences. Restrictions on the utilization of metal impetuses, cruel response conditions, costly reagents, monotonous workup, low yield, extended response time, and natural peril are instances of engineered rules.

Due to the previously mentioned challenges and our anxiety about harmless to the ecosystem techniques, most researchers have been charmed by the quest for straightforward, effective, and harmless to the ecosystem ways to deal with support natural responses in green conditions. Given the prior worries and our goal to create polysubstituted quinolines, reading on naturally safe impetuses for the right blend of nitrogen heterocyclic buildings under green conditions is crucial. The utilization of a non-metallic natural color, fluorescein, in the previously mentioned photochemical blending process is given another job in this review. Photoinduced states delivered by fluorescein have been displayed to work as an impetus for photochemically revolutionary producing polysubstituted quinolines. Apparent light guides the Friedländer hetero-annulation [58] of 2-aminoaryl ketone and α-methylene carbonyl compound in ethanol at room temperature and in an air climate. This is a fruitful one-pot response that was completed in an exceptionally proficient, unobtrusive, and direct way.

## Experimental

### General technique

A combination of 2-aminoaryl ketone (**1**, 1.0 mmol) and α-methylene carbonyl compound (**2**, 1.5 mmol) in EtOH (3 mL) was added fluorescein (0.5 mol%) and mixed at encompassing temperature under white LED (12 W) light. Attention was utilized to follow the response’s turn of events, with the eluent being *n*-hexane/ethyl acetate (3:2). The subsequent material was screened and washed with water after the response, and the rough strong was solidified again from ethanol to create the unadulterated substance without extra purging. If we could manufacture the aforementioned compounds using gram scale methods we would want to test if we could scale up to the level required for pharmaceutical process R&D. One experiment used 50 mmol of 2-aminobenzophenone and 75 mmol of acetylacetone. Using a typical filtration technique, the product was collected after only 8 min of the reaction. This material has a ^1^HNMR spectrum that suggests that it is spectroscopically pure. The products were ordered after spectroscopic information was analyzed. The products were ordered in the wake of looking at spectroscopic information (^1^HNMR). ^1^HNMR files for compounds **3c** and **3k** are provided in the Additional file 1.

## Results and discussion

**Fig. 1:**
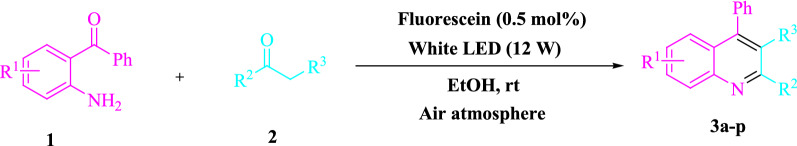
Synthesis of polysubstituted quinolines

To start, Table 1 sums up the consequences of a review into the superior reactivity of 2-aminobenzophenone (1.0 mmol) and dimedone (1.5 mmol) in EtOH (3 mL) after the light at room temperature. A follow measure of **3a** was found at room temperature for 45 min without the utilization of a photocatalyst (Additional file 1: Table S2). Fluorescein, Na_2_ eosin Y, phenanthrenequinone, erythrosin B, alizarin, rose Bengal, 9*H*-xanthen-9-one, acenaphthenequinone, riboflavin, xanthene, and rhodamine B were explored under comparative circumstances. In yields going from 48–96%, the improvement of this occasion and the formation of the matching item **3a** were seen agreeably. Fluorescein outflanked other organophotocatalysts in this cycle, as per our discoveries. The yield was expanded to 96% by adding 0.5 mol% fluorescein. What’s more, item yields in DMF, toluene, THF, DMSO, CHCl_3_, and CH_2_Cl_2_ were low (Additional file 1: Table S3). The yield and pace of the response rose as the response progressed in H_2_O/EtOH, H_2_O, MeOH, solvent-free, CH_3_CN, EtOAc. In EtOH, the response went extremely well, giving 96% under similar circumstances. The yield was assessed under different lighting conditions and displayed to rise to some degree because of white light. A control exploration uncovered that even without a light source, a hint of the synthetic could be recognized. The revelation underlines the significance of fluorescein and apparent light in the item’s turn of events. Also, the best conditions were found by fluctuating the white LED illumination powers. Additional file 1: Table S3 shows that when white 12 W LED illumination was utilized, the best outcomes were gotten. This approach can be utilized on different substrates, as exhibited in Table 2 and Fig. 1. (More data is provided in Additional file 1: Tables S2 and S3).

**Table 1:** Photocatalyst, solvent, and visible light optimization table


Entry	Photocatalyst	Light Source	Solvent (3 mL)	Time (min)	Isolated yields (%)
1	Fluorescein (0.2 mol%)	White light (12 W)	EtOH	15	74
**2**	**Fluorescein (0.5 mol%)**	**White light (12 W)**	**EtOH**	**10**	**96**
3	Fluorescein (1 mol%)	White light (12 W)	EtOH	10	96
4	Fluorescein (0.5 mol%)	White light (10 W)	EtOH	10	87
5	Fluorescein (0.5 mol%)	White light (18 W)	EtOH	10	96
6	Fluorescein (0.5 mol%)	White light (12 W)	H_2_O	10	81
7	Fluorescein (0.5 mol%)	White light (12 W)	H_2_O/EtOH (1:1)	10	86

Reaction condition: at rt, 2-aminobenzophenone (1.0 mmol) and dimedone (1.5 mmol) in different fluorescein molars, and a variety of solvents and white LED illumination powers were used

**Table 2:**
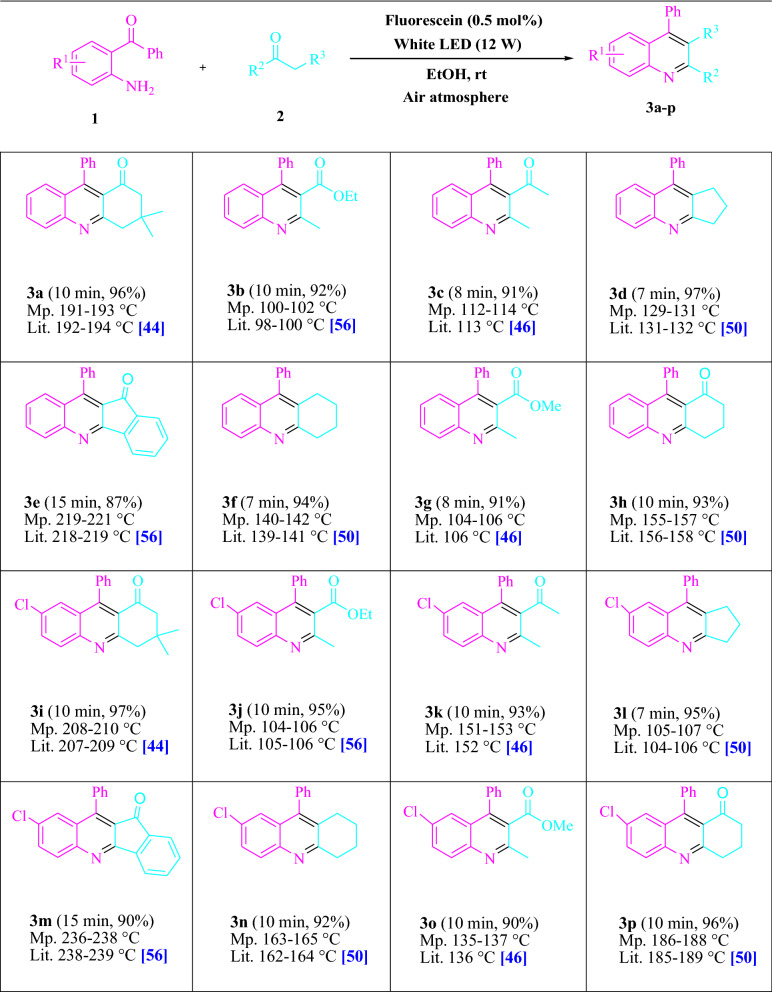
Photocatalyst for the production of polysubstituted quinolines using photoexcited fluorescein

Table 3 likewise remembers data for turnover number (TON) and frequency of turnover (TOF). The higher the TON and TOF mathematical qualities are, the less catalyst is used and the higher the yield, and the catalyst becomes more effective as the value grows.

**Table 3:** Values of TON and TOF calculated

Entry	Product	TON	TOF	Entry	Product	TON	TOF
1	**3a**	192	19.2	9	**3i**	194	19.4
2	**3b**	184	18.4	10	**3j**	190	19
3	**3c**	182	22.7	11	**3k**	186	18.6
4	**3d**	194	27.7	12	**3l**	190	27.1
5	**3e**	174	11.6	13	**3m**	180	12
6	**3f**	188	26.8	14	**3n**	192	19.2
7	**3g**	182	22.7	15	**3o**	180	18
8	**3h**	186	18.6	16	**3p**	192	19.2

**Fig. 2:**
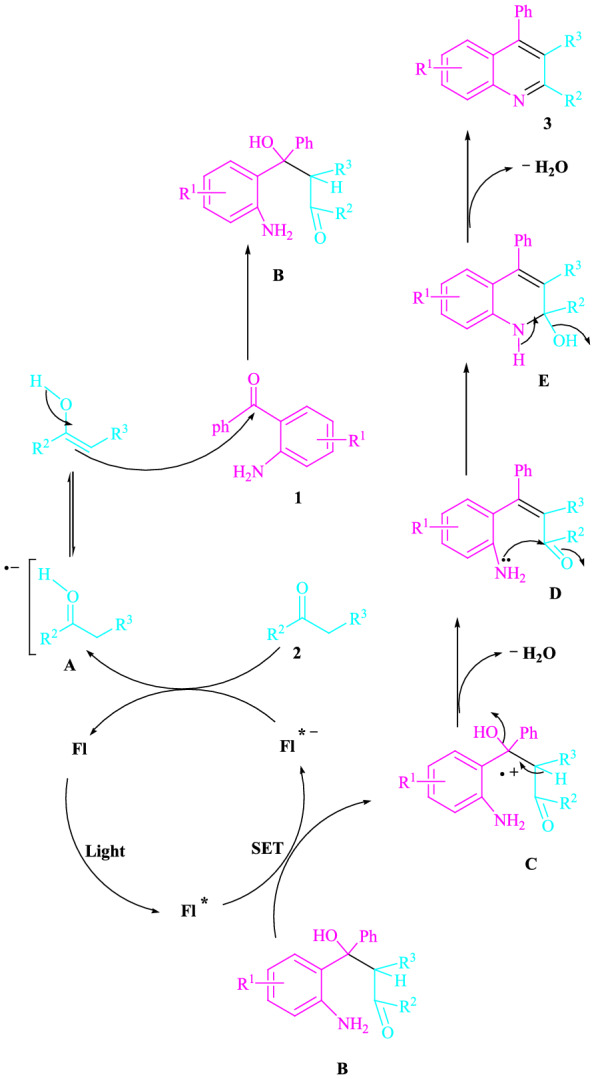
A mechanistic technique for the synthesis of polysubstituted quinolines has been proposed. Fl: fluorescein

The preferred mechanism is denoted in Fig. 2. The visible light can be changed in part by the application of more energy to speed up this reaction. This fluorescein, according to earlier studies [14], uses visible light as a source of renewable energy to build acceptable catalytic methods employing a single-electron transfer (SET) pathway. Through an energy transfer (EnT) between Fl^*−^ and -methylene carbonyl compound **2** regenerates the ground-state fluorescein and the intermediate **A**. When this radical anion **A** is nucleophilically added to 2-aminoaryl ketone **1**, a reactive intermediate **B** is formed. Then, a SET pathway promotes visible light-triggered fluorescein^*^, which produces the cation radical **C**. The cyclized dehydrated is then added for a total of **3**.

The photoredox cycle is started out whilst dye inside the ground state is irradiated with visible light to provide the high-energy excited state of dye (Dye^*^). The system of seen mild photoredox catalysis is supplied by the use of separate paths from dye inside the excited state (Dye^*^). Within the presence of a sacrificial electron acceptor, Dye^*^ reductive’s belongings can be hired. In different phrases, Dye^*^ leads the unconventional cation species of Dye as an electron donor. Within the presence of a sacrificial electron donor, Dye^*^ also works as an electron acceptor [59].

## Conclusion

At long last, the photoinduced conditions of fluorescein-determined go about as an impetus for photochemically combining polysubstituted quinolines by extremist Friedländer hetero-annulation of 2-aminoaryl ketone and α-methylene carbonyl compound in EtOH at a surrounding temperature in an air environment. This study lays out a clever capability for photochemically combining fluorescein, a non-metallic natural color that is economically accessible and reasonable while creating great outcomes, accelerating the interaction, and achieving a high iota economy. This is an effective one-pot response that was acted in an exceptionally proficient, moderate, and direct way.

## Supplementary Information

**Additional file 1.** **Figure S1.** ^1^HNMR Spectrum of compound of **3c**. Figure S2. ^1^HNMR Spectrum of compound of **3K**. **Table S1.** Comparison of ^1^HNMR data. **Table S2.** Photocatalyst optimization table. **Table S3.** Solvent and visible light optimization table.

## Data Availability

All data generated or analyzed during this study are included in this published article and its Supplementary Information file.
