# Supplementary material for: Visible-light-driven radical Friedländer hetero-annulation of 2-aminoaryl ketone and α-methylene carbonyl compound via organic dye fluorescein through a single-electron transfer (SET) pathway
Source: BMC Chem. 2022 Dec 15;16(1):116. doi: 10.1186/s13065-022-00910-1 (PMC9753410; doi:10.1186/s13065-022-00910-1)
Supplement: Supplementary file 1 — Additional file 1. Figure S1. 1HNMR Spectrum of compound of 3c. Figure S2. 1HNMR Spectrum of compound of 3K. Table S1. Comparison of 1HNMR data. Table S2. Photocatalyst optimization table. Table S3. Solvent and visible light optimization table. [file 13065_2022_910_MOESM1_ESM.pdf]

Supporting Information

Visible-light-driven radical Friedländer hetero-annulation of 2-aminoaryl ketone and α-methylene carbonyl compound via organic dye fluorescein through a single-electron transfer (SET) pathway

Farzaneh Mohamadpour *

School of Engineering, Apadana Institute of Higher Education, Shiraz, Iran

* Corresponding author. mohamadpour.f.7@gmail.com

1-(2-Methyl-4-phenylquinolin-3-yl)ethanone (3c)

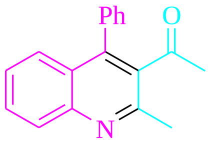

3C

Yield: 91%; M.p. 112-114 °C; ^{1}HNMR (300 MHz, $CDCl_3$): 2.03 (3H, s, CH_3), 2.65 (3H, s, CH_3), 7.39–7.46 (6H, m, ArH), 7.53 (1H, d, *J* = 7.2 Hz, ArH), 7.64–7.66 (1H, t, *J* = 7.2 Hz, ArH), 8.02 (1H, d, *J* = 8.4 Hz, ArH).

1-(6-Chloro-2-methyl-4-phenylquinolin-3-yl)ethanone (3k)

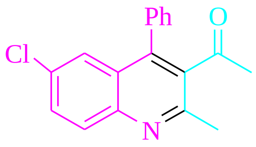

3K

Yield: 93%; M.p. 151-153 °C; ^{1}HNMR (300 MHz, $CDCl_3$): 2.01 (3H, s, CH_3), 2.69 (3H, s, CH_3), 7.36–7.41 (2H, m, ArH), 7.50–7.59 (5H, m, ArH), 8.04 (1H, d, *J* = 8.4 Hz, ArH).

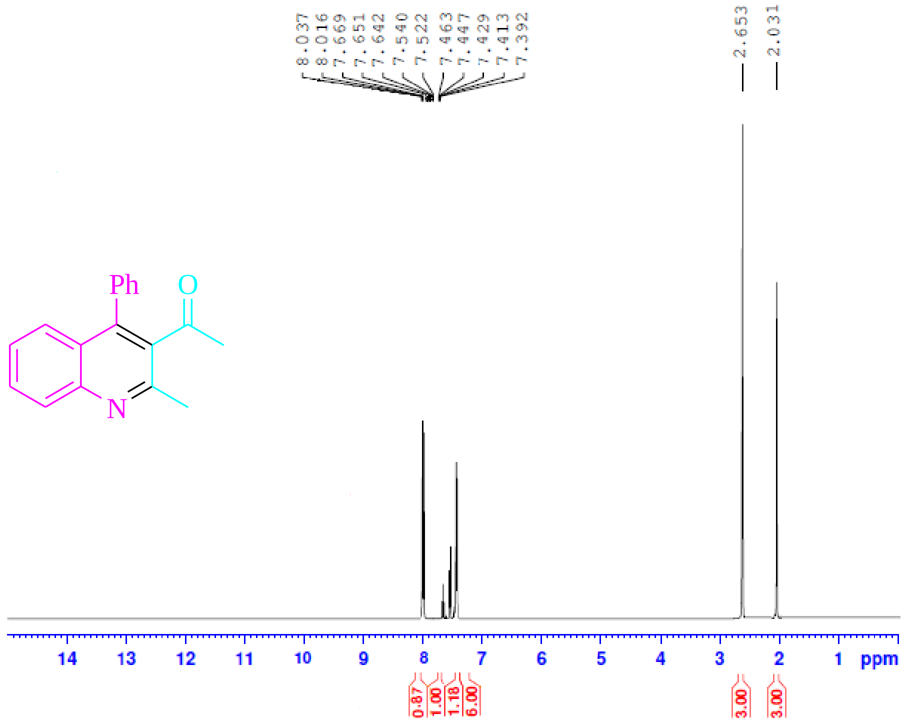

Figure S1. ^{1}HNMR Spectrum of compound (300 MHz, $CDCl_3$) of **3c**

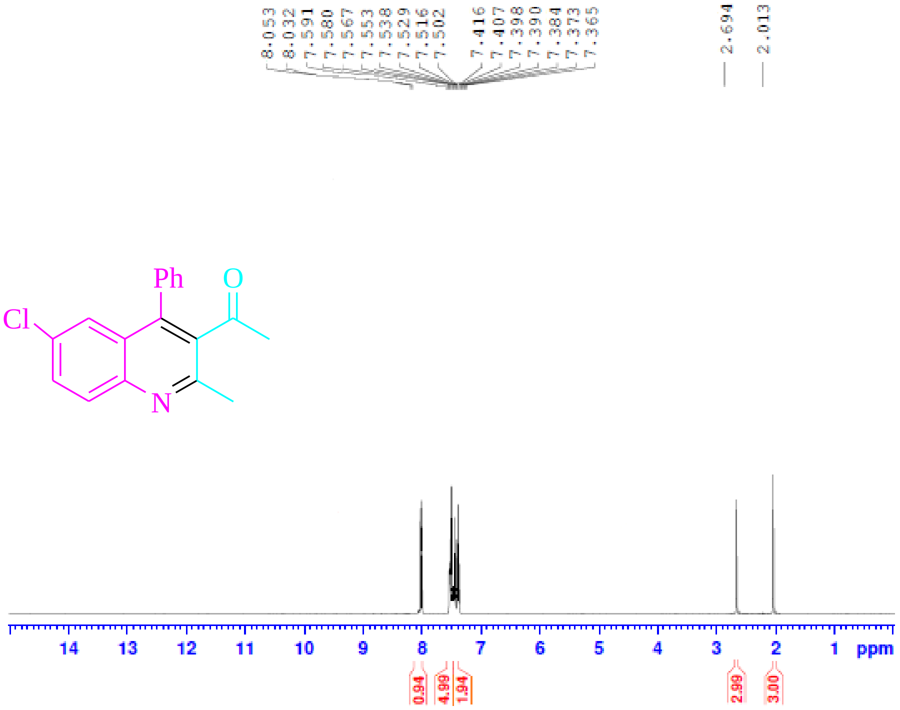

Figure S2. ^{1}HNMR Spectrum of compound (300 MHz, $CDCl_3$) of **3k**

Table S1. Comparison of ^{1}HNMR data.

Entry	Product	H Shift (Found)	H Shift (Lit.)	Reference
1	**3c**	2.03 (3H, s, CH_3)	1.96 (3H, s)	[55]
		2.65 (3H, s, CH_3)	2.68 (3H, s)	
		7.39–7.46 (6H, m, ArH)	7.35–7.50 (6H, m)	
		7.53 (1H, d, *J* = 7.2 Hz, ArH)	7.60 (1H, d, *J* = 7.9 Hz)	

		7.64–7.66 (1H, t, J= 7.2 Hz, ArH)<br>8.02 (1H, d, J = 8.4 Hz, ArH)	7.68–7.70 (1H, t, J = 7.9 Hz)<br>8.05 (1H, d, J = 8.0 Hz)	
2	**3k**	2.01 (3H, s, CH_3)<br>2.69 (3H, s, CH_3)<br>7.36–7.41 (2H, m, ArH)<br>7.50–7.59 (5H, m, ArH)<br>8.04 (1H, d, J = 8.4 Hz, ArH)	2.00 (3H, s)<br>2.68 (3H, s)<br>7.33–7.35 (2H, m)<br>7.53–7.66 (5H, m)<br>8.00 (1H, d, J = 8.9 Hz)	[48]

Table S2. Photocatalyst optimization table[a]

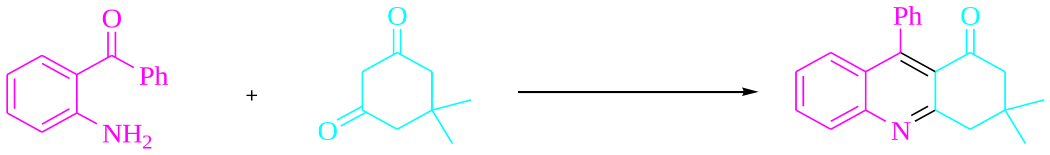

Entry	Photocatalyst	Solvent (3 mL)	Time (min)	Isolated Yields (%)
1	—	EtOH	45	trace
2	Na_2 eosin Y (0.5 mol%)	EtOH	10	81
3	Phenanthrenequinone (0.5 mol%)	EtOH	10	62
4	Erythrosin B (0.5 mol%)	EtOH	10	64
5	Alizarin (0.5 mol%)	EtOH	10	51
6	Rose bengal (0.5 mol%)	EtOH	10	65
7	9*H*-Xanthen-9-one (0.5 mol%)	EtOH	10	54
8	Acenaphthenequinone (0.5 mol%)	EtOH	10	48
9	Riboflavin (0.5 mol%)	EtOH	10	66

10	Xanthene (0.5 mol%)	EtOH	10	57
11	Rhodamine B (0.5 mol%)	EtOH	10	73

[a]Reaction condition: at rt, 2-aminobenzophenone (1.0 mmol) and dimedone (1.5 mmol) in EtOH, a white LED (12 W), and a variety of photocatalysts were used.

Table S3. Solvent and visible light optimization table[a]

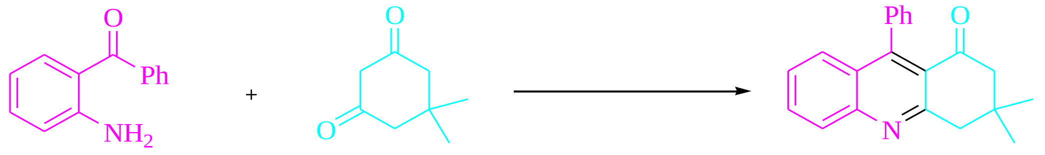

Entry	Light Source	Solvent (3 mL)	Time (min)	Isolated Yields (%)
1	White light (12 W)	H_2O/EtOH (2:1)	10	82
2	White light (12 W)	H_2O/EtOH (1:2)	10	89
**3**	**White light (12 W)**	**EtOH**	**10**	**96**
4	White light (12 W)	MeOH	20	62
5	White light (12 W)	—	25	65
6	White light (12 W)	CH_3CN	10	67
7	White light (12 W)	EtOAc	15	64
8	White light (12 W)	DMF	20	43
9	White light (12 W)	Toluene	25	48
10	White light (12 W)	THF	20	39
11	White light (12 W)	DMSO	20	46
12	White light (12 W)	$CHCl_3$	50	32

13	White light (12 W)	CH_2Cl_2	50	36
14	Blue light (12 W)	EtOH	10	91
15	Green light (12 W)	EtOH	10	86
16	__	EtOH	50	<5

[a]Reaction condition: at rt, 2-aminobenzophenone (1.0 mmol) and dimedone (1.5 mmol) were added to fluorescein (0.5 mol%).

References

[48] Wu J, Xia HG, Gao K. Molecular iodine: a highly efficient catalyst in the synthesis of quinolines via Friedländer annulation. Organic & Biomolecular Chemistry. 2006; 4: 126-9.

[55] Yadav JS, Reddy BS, Sreedhar P, Rao RS, Nagaiah K. Silver phosphotungstate: A novel and recyclable heteropoly acid for Friedländer quinoline synthesis. Synthesis. 2004; 2004: 2381-5.
